# Micro- and mesoporous solids: From science to application

**DOI:** 10.3762/bjnano.2.85

**Published:** 2011-11-30

**Authors:** Jörg J Schneider

**Affiliations:** 1 Department of Chemistry, Eduard-Zintl-Institute, Inorganic Chemistry, Technische Universität Darmstadt, Petersenstr. 18, 64287 Darmstadt, Germany

The concept of a porous solid might sound like a term of contradiction as solids are typically regarded as a dense and compact state of matter. Of course this is obviously not so, and zeolites are probably the most prominent representatives of such solids, known for already more than 250 years, and have been used in technical applications for more than 50 years in industry. 200 different structural types of zeolites have already been listed, with this number increasing each year. However, the pore sizes of zeolites are restricted to well below 1 nm (for faujasite), which in turn restricts the scope of the chemistry that is possible inside the pores, typically to the molecular level.

**Figure 1:**
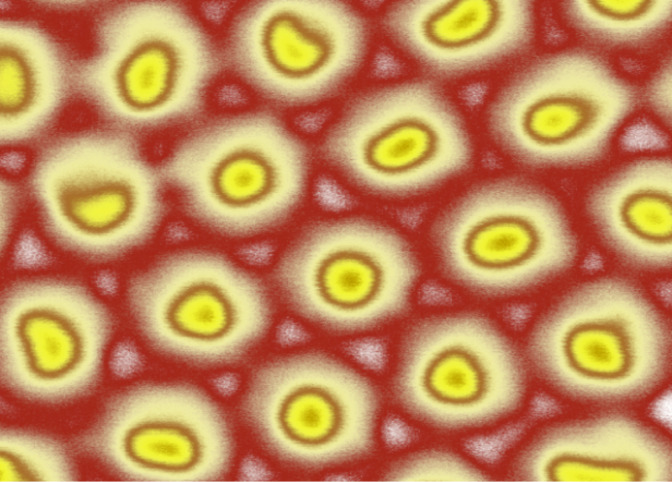
Artificially colorized view of a hexagonally ordered cell structure of mesoporous alumina. The pores are shown in yellow and are ca. 40 nm in diameter. The red color grading represents the typically different densities of the alumina cells surrounding the pores, which is due to the synthesis process. As with other mesoporous materials, the inner surface of the pores can be chemically modified to tune the surface chemistry and thus the functional properties.

However, in the last two decades especially, mesoporous materials having pore dimensions between 2 and approximately 50 nm have emerged, and this group has established itself as an important class of solid-state materials with a huge and still constantly growing number of new congeners with pores on the nano- to mesoscale. These can be broadly classified into inorganic, organic and metal–organic types. Nevertheless, hybrids of these compositions have even been realized, extending the diversity in the chemical composition of such mesoporous solids further still. Besides their different chemical composition, the pore morphology, geometry and pore dimensions make these materials outstanding with respect to, e.g., catalytic reaction processes, in the area of sensorics, photonics and gas storage (Figure 1). In the realm of gas storage, mesoporous metal–organic frameworks (MOFs) appeared on the scene a couple of years ago and have quickly emerged as most-promising highly meso- and macroporous materials exhibiting enormous pore volumes with inner surface areas comparable with bare nanoparticles and thus coming close to the ultimate adsorption limit for solid materials. Consequently they have paved the way for the design of new materials, e.g., for the storage of fuel molecules, such as H_2_ and CH_4_ as well as other technologically and environmentally important small molecules. Besides the prospective applications of mesoporous materials in these and other areas, one of the other main focus points of research into these solids is to achieve a basic understanding of what happens inside the porous framework of such a solid on the molecular, and nano- and mesocopic level, in a hierarchical order, during adsorption, desorption and chemical reactions. Alongside the development of experimental methods to unravel the details of the adsorption sites and dynamics, a theoretical understanding, e.g., employing state of the art theoretical modeling techniques, is certainly necessary. The development of synthetic concepts for the formation of two-dimensional, layered, porous structures, e.g., by swelling and delamination followed by film-formation techniques, is another avenue of basic research into exciting mesoporous materials, and the application of such mesoporous layered materials to adsorption and catalysis can certainly be envisaged in the near future.

In this Thematic Series the interested reader will find a compilation of recent experimental findings relating to mesoporous inorganic solids, covering aspects of synthesis as well as the functional properties of such materials in catalysis and sensing.

Jörg J. Schneider

Darmstadt, November 2011

